# Comparative Efficacy of Two Hemostatic Agents in Post-Extraction Bleeding Control Following Mandibular Third Molar Surgery: A Randomized Clinical Trial

**DOI:** 10.3390/jfb16090305

**Published:** 2025-08-22

**Authors:** Giovanna Pesce, Suelen Cristina Sartoretto, Rodrigo Figueiredo de Brito Resende, Madelaine Torres da Silva, Jose Mauro Granjeiro, Massimo Del Fabbro, Carlos Fernando Mourão, Monica Calasans-Maia

**Affiliations:** 1 Post-Graduation Program in Dentistry, Fluminense Federal University, Niteroi 24220-140, Brazil; dragiovannapesce@gmail.com (G.P.); madetorres.mt@gmail.com (M.T.d.S.); 2 Oral Surgery Department, Fluminense Federal University, Niteroi 24220-140, Brazil; susartoretto@hotmail.com (S.C.S.); rodrigoodonto21@hotmail.com (R.F.d.B.R.); 3 Clinical Research Laboratory, Dentistry School, Universidade Federal Fluminense, Niteroi 24220-140, Brazil; jmgranjeiro@gmail.com (J.M.G.); monicacalasansmaia@gmail.com (M.C.-M.); 4 National Institute of Metrology, Quality and Technology (INMETRO), Duque de Caxias 25250-020, Brazil; 5 Department of Biomedical, Surgical and Dental Sciences, Università Degli Studi di Milano, 20122 Milan, Italy; massimo.delfabbro@unimi.it; 6 Fondazione IRCCS Ca’ Granda Ospedale Maggiore Policlinico, 20122 Milan, Italy; 7 Department of Basic and Clinical Translational Sciences, Tufts University School of Dental Medicine, Boston, MA 02111, USA

**Keywords:** lower third molar extraction, local hemostasis, soft tissue

## Abstract

Adequate bleeding control is crucial in surgical procedures. Surgifoam and Hemospon are absorbable hemostatic sponges made from lyophilized porcine gelatin, commonly used in oral surgery. This clinical study aimed to evaluate bleeding control, soft tissue healing, and postoperative pain in dental sockets after mandibular third molar extractions filled with Surgifoam and Hemospon. Twenty-five volunteers requiring extractions of both left and right lower third molars participated in this randomized, double-blind, split-mouth study. After extraction, each socket was randomly filled with Hemospon (test group) or Surgifoam (control group). Postoperative pain was assessed using the Visual Analog Scale (VAS) on days 1, 2, 3, and 7. Bleeding at 30 and 60 min (Souto and Mühlemann scales) and soft tissue healing at 7 and 14 days (Brancaccio scale) were evaluated using Fisher’s exact test. Bleeding scores at 30 min predominantly showed no bleeding (score 0) in 80% of participants using Surgifoam, compared to 60% in the Hemospon group. No significant differences in bleeding were observed between groups (*p* > 0.05), and both showed a similar reduction over time. Soft tissue healing was revealed at 14 days complete healing (score 0) in 90% of participants in both groups. No significant differences between Hemospon^®^ and Surgifoam^®^ were observed (*p* > 0.05). Postoperative pain evaluation showed highly variable scores on the first day (median: 2; range: 1–6) for both Surgifoam^®^ and Hemospon^®^. By the seventh day, pain levels significantly reduced (median: 0), with no significant differences observed between the groups at any time point (*p* > 0.05). In conclusion, the results of this study suggest that Hemospon and Surgifoam are equally effective in bleeding control, healing, and pain control after third molar surgery. This research aims to guide surgeons on the clinical aspects of using these specific hemostatic sponges in post-extraction procedures for posterior molars and seeks to direct future clinical studies involving these materials.

## 1. Introduction

Lower third molar extractions are the most common surgeries performed by dental surgeons. These extractions often result in complications such as periodontal bone defects compromising the distal surface of the remaining second molars, bleeding, pain, trismus, alveolitis, and edema [1,2,3].

Changes in the bleeding pattern in some patients may be related to the position of tooth impaction, resulting in surgical difficulty. More invasive surgical techniques, such as ostectomy and odontotomy, may be necessary depending on how unfavorable the tooth position in the alveolar socket is [4,5].

A series of clinical-surgical strategies has been used to avoid or reduce postoperative complications. Some methods have been developed to improve periodontal defects and encourage healing, such as different flap designs for access and extraction of third molars for better root preservation of the second molar and adjacent bone tissue [6], suturing techniques that further improve tissue healing [4,5,6,7], bone grafts with or without membranes (absorbable or non-absorbable) [8,9], platelet concentrates to fill the dental socket [10,11], laser therapy [12], and implantation of hemostatic collagen sponges in the socket after tooth extraction [13].

Hemostasis is essential for reducing postoperative complications. In recent years, traditional hemostatic materials have been widely used in clinical dental practice. Among them, collagen sponges are prominent [14]. Collagen sponges were used to reduce local post-surgical bleeding and thus promote more efficient healing of soft tissues, also helping control postoperative pain. Understanding the blood clotting mechanism is the premise for selecting the hemostatic biomaterial.

Therefore, it is notable that a good hemostatic agent must facilitate the clotting process. A suitable hemostatic biomaterial must have the following characteristics: biocompatibility, non-toxicity, non-antigenicity, adequate elasticity, good gas permeability, water permeability, low probability of infection, and fast hemostasis [15].

Recent advances in biotechnology have led to the development of absorbable hemostatic agents for dental surgery, including gelatins, microfibrillar collagen, oxidized cellulose, thrombin, biological glues, and other combined agents [16,17]. Gelatin, introduced in 1945, is derived from animal collagen (porcine, sheep, or equine) and is available in sponge, powder, or saline solution forms [18].

The collagen sponge acts as an extracellular matrix, favoring the migration of osteoblasts, stabilizing blood clots, facilitating the healing of soft tissues, and helping protect wounds and bone reconstruction [19].

Surgifoam is a porous hemostatic sponge comprising freeze-dried gelatin of porcine origin, which is sterile, insoluble in water, malleable, and absorbable. The mode of action of hemostatic sponges remains unclear; however, it is believed to involve the formation of a mechanical three-dimensional matrix that facilitates coagulation without affecting the blood clotting mechanism [20,21,22]. When applied to the mucosa, Surgifoam completely liquefies in 2 to 5 days and is reabsorbed after 4 to 6 weeks. Gelatin is hygroscopic, absorbing many times its weight in water. It promotes the concentration of platelets and clotting factors and expands the gelatin, providing additional mechanical hemostatic action through compression [23,24].

Hemospon also comprises gelatin (100% collagen) of porcine origin, indicated for local hemostasis, and it has the same mechanism of action as Surgifoam. A previous study conducted in rats evaluated sockets filled with Gelfoam compared to Hemospon. It concluded that both hemostatic agents are similar in terms of biological events occurring in the gingival mucosa and in alveolar repair throughout the entire experimental period [24]. The difference between the two sponges lies in their manufacture, since they contain the same compounds.

A recent randomized controlled clinical study concluded that the implantation of a collagen sponge of porcine origin (Ateloplug) after the extraction of impacted lower third molars reduced initial postoperative complications and improved the initial healing of soft and periodontal tissues compared to sockets without filling (blood clot) [13].

Previous research has established the safety of hemostatic agents; however, their clinical properties related to bleeding control, tissue healing, and postoperative pain management remain insufficiently demonstrated. These properties need to be clarified and provided as a foundation for future research [13,14].

The present study aimed to clinically and comparatively evaluate the rate of bleeding, soft tissue healing, and postoperative pain after the use of two hemostatic agents, Surgifoam and Hemospon, which were topically implanted after third lower molar extraction in 25 research participants in a randomized, controlled, and blinded study. Both sponges are commercially available, and their safety has been established. However, they have not yet been tested in post-third molar extraction sites in a randomized design, which is crucial for assisting surgeons in selecting the most appropriate material for these surgical cases.

## 2. Materials and Methods

### 2.1. Ethical Considerations

This project was approved by the Research Ethics Committee (CEP) of the Faculty of Medicine of the Universidade Federal Fluminense (4,718,619), and the trial was registered in the Brazilian Clinical Trials Registry (REBEC—registration number: RBR-29fjs5r, 18 August 2025). The trial was conducted in accordance with the Declaration of Helsinki.

### 2.2. Materials

Two hemostatic sponges were used for implantation in the dental sockets of lower third molars:

- Control: Surgifoam (Ethicon, Johnson & Johnson, New Brunswick, NJ, USA).

Surgifoam is an absorbable, sterile, water-insoluble, malleable porcine gelatin sponge saturated with sterile sodium chloride solution.

- Test: Hemospon (Maquira Laboratory, Maringá, Paraná, Brazil).

Hemospon is a lyophilized porcine gelatin hemostatic sponge with hemostatic healing action. It is non-toxic and non-pyrogenic.

### 2.3. Research Participants

Twenty-five research participants in need of a bilateral extraction (50 dental sockets) were recruited and divided into two experimental groups: 25 sockets filled with Hemospon, and 25 sockets filled with Surgifoam. Extraction was indicated after clinical and radiographic examination by a professional specializing in oral and maxillofacial surgery, who was not part of this project. The clinical and surgical procedures were conducted at the Associated Laboratory of Clinical Research in Dentistry (LPCO) of the Dentistry School of Universidade Federal Fluminense. All participants received detailed verbal and written information about the nature, objectives, potential risks, and benefits of the study in a language they could easily understand. Each participant was given the opportunity to ask questions and discuss concerns with the investigators prior to consenting. Written informed consent was obtained before participation, and participants retained copies for their records. Confidentiality was ensured by de-identifying all participant data and securely storing it in accordance with the principles of Good Clinical Practice and the ethical guidelines outlined in the Declaration of Helsinki.

### 2.4. Sample Size Calculation

The sample size for this study was determined using data from the study by Sybil et al., (2020) [25], which reported a standard deviation of 0.55 for the control group with a sample size of n = 25. Given the nature of the bleeding index, which ranges from 0 to 3, and each unit representing a distinct level of bleeding, a more conservative sample size was adopted.

The equivalence value (d) was set to 1, reflecting the anticipated minimal clinically important difference between the hemostats Surgifoam and Hemospon. This effect size assumption was based on clinical relevance, as a difference of 1 unit on the bleeding index is considered meaningful in a clinical context.

Using the Sealed Envelope online platform, the calculated sample size was determined to be 22 participants for each experimental group, with a significance level of 5% and a power (1-β) of 90%. To account for a potential dropout rate of 10%, the final recruitment target was adjusted to 25 participants per group for this split-mouth study design, ensuring adequate power to detect differences between the hemostats.

### 2.5. Physical and Psychological Inclusion Criteria

The physical criteria included research participants with bilateral third molars indicated for extraction, aged between 18 and 40 at the time of recruitment.

The psychological criteria for participation in the project comprised patients with no history of anxiety, mood, eating, or psychotic disorders that could compromise their participation and collaboration in the study. These were evaluated by completing an anamnesis form.

### 2.6. Exclusion Criteria

All research participants who smoked, were dependent on alcohol, or had systemic diseases (e.g., diabetes, blood dyscrasias) were excluded from the study.

### 2.7. Evaluation Periods

All research participants were evaluated before extraction, after 30 and 60 min of extraction, and after 7 and 14 days. On the day of the surgeries, a photograph was taken of the region of the extracted teeth, as well as a panoramic X-ray of the jaws. All research participants were instructed to read the Image Use Assignment Term and sign it to confirm agreement.

### 2.8. Study Design

The study adopted a randomized and controlled split-mouth design. Immediately before the start of the extractions, the side of the hemostatic sponge implantation was randomized using a coin with tails on the right side and heads on the left.

The advantage of the split-mouth study compared to parallel studies is the need for a smaller sample size [26,27]. In this study format, it is possible to minimize intra-individual variables since research participants serve as their own controls and interfere equally with treatments. This reduction in intra-individual variability increases the power of the study [28,29].

### 2.9. Blinding and Randomization

All surgeries were performed by the same operator with the same pre-, trans-, and postoperative protocol, and the examiner responsible for postoperative assessments was not present in any surgeries, blinding the study to the assessment. Furthermore, no participants were informed about which socket received the Hemospon or Surgifoam to blind the research participants; thus, a double-blind study was conducted. Randomization was performed through the flipping of a coin, where each side represented an experimental group. To address potential imbalances in group distribution, participants were stratified prior to randomization based on relevant prognostic factors (e.g., age, gender, and baseline clinical parameters). The allocation sequence was concealed until participants were enrolled and assigned to interventions.

Additionally, baseline characteristics were compared between groups after randomization using appropriate statistical tests to verify balance, and covariate adjustments were applied during statistical analysis if necessary. These measures ensured that any bias or imbalance introduced by the randomization method was minimized. The sides—the right or left dental sockets—were distributed into two groups: Hemospon (n = 25) and Surgifoam (n = 25).

### 2.10. Surgical Procedures

**Figure 1:**
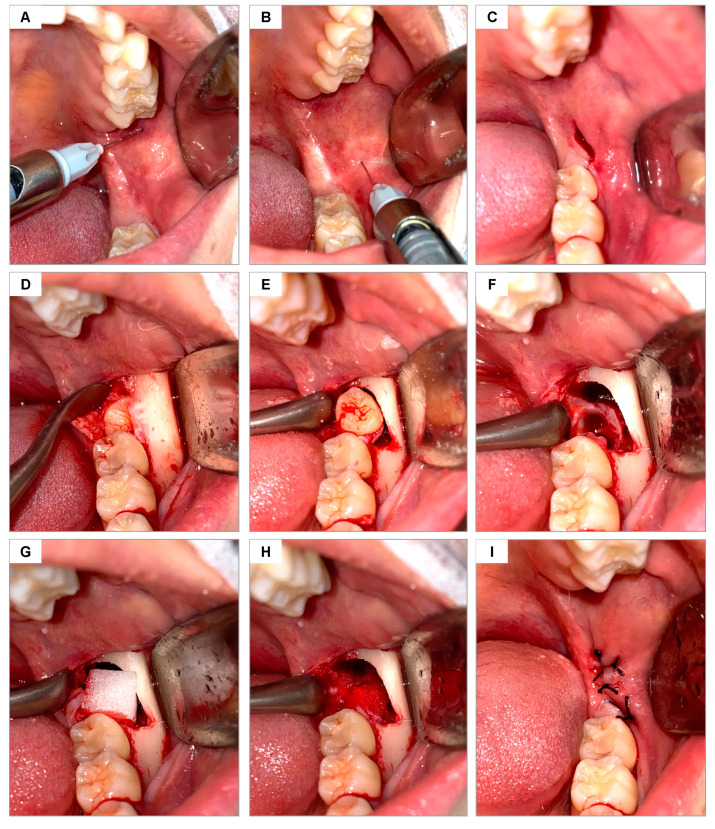
Surgical sequence of the methodology used for dental extractions: (**A**,**B**) local anesthesia for the blockade of the inferior alveolar/lingual and buccal nerves, respectively; (**C**) incision to create an envelope flap; (**D**) exposure of the cortical bone and the occluso-lingual surface of the tooth to be extracted; (**E**) luxation of the tooth; (**F**) alveolar bone after tooth extraction; (**G**) hemostatic sponge being placed inside the dental socket; (**H**) sponge adjusted inside the socket; (**I**) suturing.

The area was cleaned by rinsing the mouth with 0.12% chlorhexidine digluconate (Periogard Colgate, Rio de Janeiro, Brazil) for 1 min and extraorally using 4% chlorhexidine soap (Riohex Rioquímica, Duque de Caxias, Brazil). The sterile surgical field was then positioned, and local anesthesia was administered using a carpule-type syringe (Quinelato, Schobell Industrial Ltda, São Paulo, SP, Brazil) to block the inferior alveolar, lingual, and buccal nerves, using articaine 4% with epinephrine 1:100,000 (DFL Indústria e Comércio, Rio de Janeiro, RJ, Brazil) (Figure 1A,B). The same surgical approach was used in all surgeries, following the technique described by Hupp [30] for open extractions of lower molars. After confirming the absence of pain, the surgical envelope flap was performed using a #3 scalpel handle (Bard Park, Quinelato, Schobell Industrial Ltda, São Paulo, SP, Brazil) and #15 blade (Solidor, Lamelid, Osasco, SP, Brazil) (Figure 1C). The tissue was detached using the Molt no. 9 detacher (Quinelato, Schobell Industrial Ltda, São Paulo, SP, Brazil) (Figure 1D). Ostectomy and odontosection, when necessary, were performed with surgical drill no. 6 (Angelus Ltda, Londrina, PR, Brazil) and frustoconical drill no. 702 (Angelus Ltda, Londrina, PR, Brazil) under constant irrigation with sterile 0.9% physiological saline solution. The dislocation of the dental element was performed using an Apexo lever no. 3 (Quinelato, Schobell Industrial Ltda, São Paulo, SP, Brazil) (Figure 1E) for subsequent extraction (Figure 1F). Once the extraction was completed, the alveoli were delicately curetted with a Lucas curette no. 4 (Quinelato, Schobell Industrial Ltda, São Paulo, SP, Brazil) and irrigated with 0.9% physiological saline solution.

Based on the draw performed, one alveolus was filled with Hemospon sponge and the other with Surgifoam sponge (Figure 1G,H). The soft tissues were sutured with Johnson 4-0 silk thread (J&J Ethicon, Jardim das Indústrias, São José dos Campos, SP, Brazil), coapting the edges with simple stitches (Figure 1I).

In both groups, the suture was performed without tension, and the research participants were instructed to perform oral hygiene by rinsing their mouths with 0.2% chlorhexidine digluconate (Perioxidin gel, Lacer, Rio de Janeiro, Brazil) twice daily, starting on the day after surgery and maintaining for 7 days. Analgesia was achieved with 750 mg of paracetamol (Sanofi Medley, Campinas, SP, Brazil), only in case of pain, and azithromycin 500 mg (Sanofi Medley Campinas, SP, Brazil), one tablet per day for 5 days, starting on the day of surgery.

The range time to perform surgeries on both sides was 45–55 min (from anesthesia to suturing on both sides).

### 2.11. Postoperative Evaluation

Consultations for postoperative evaluation were conducted by the same examiner, who was blinded regarding which side, right or left, each hemostatic sponge was placed. At the end of the surgical procedures, all research participants were kept in the Laboratory of Clinical Research in Dentistry (LPCO) for at least 1 h to check the condition of local bleeding. They were again evaluated after 1 and 2 weeks regarding the bleeding index, soft tissue healing, and the analog pain scale. Sutures were removed after 1 week.

### 2.12. Bleeding

Bleeding analysis was performed 30 and 60 min after the end of the alveolar suture and after 7 and 14 days postoperatively. Bleeding was assessed using the Souto [31,32] and Mühlemann & Son [33] scales.

**Figure 2:**
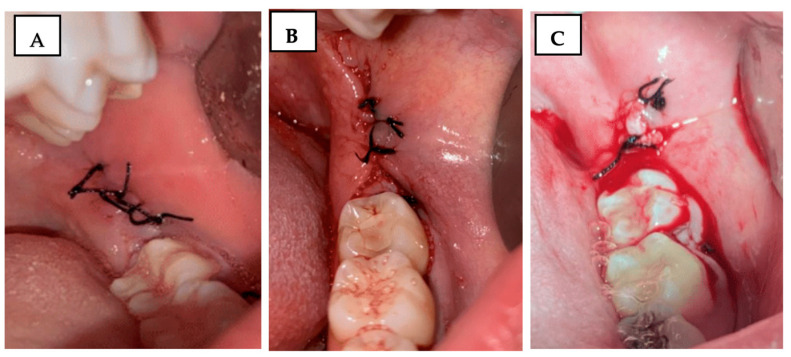
Clinical photograph illustrating the Souto scale for bleeding assessment: (**A**) score 0—no bleeding; (**B**) score 1—mild bleeding; (**C**) score 2—severe bleeding.

Using the Souto scale [31], the operated region was carefully observed using a Minnesota retractor (Quinelato, Rio Claro, SP, Brazil), and bleeding was evaluated and classified on a scale of 0 to 2 (Figure 2):

0 = No bleeding;

1 = Light bleeding, bleeding that stops spontaneously or with minimal local compression;

2 = Severe bleeding, bleeding that does not stop with the previous measures and requires continuous local compression, use of gauze soaked in antifibrinolytic, or local adrenaline.

**Figure 3:**
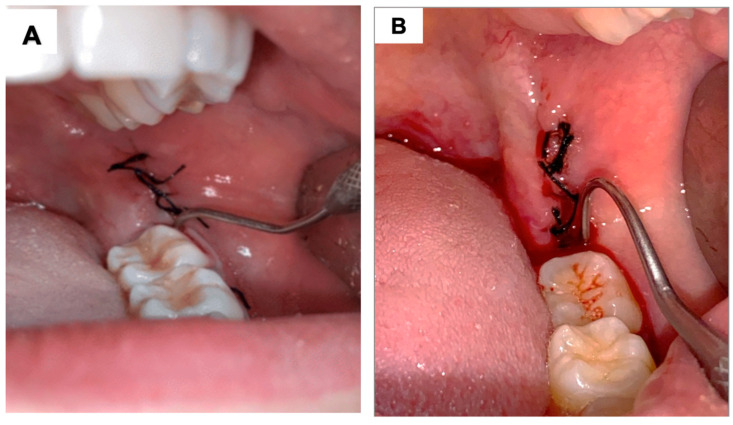
Clinical photograph illustrating the Mühlemann scale for bleeding assessment associated with probing: (**A**) represents score 0, where, after 30 s of probing the distal surface of the adjacent tooth, no bleeding occurs. (**B**) represents bleeding after probing, which can correspond to scores 1, 2, or 3, depending on the timing of the onset of bleeding.

Using the Mühlemann scale [33], the distal region of the lower second molar, adjacent to the operated area, was probed for 30 s with an exploring probe (Quinelato, Rio Claro, SP, Brazil), and the bleeding was evaluated and classified on a scale from 0 to 3 (Figure 3):

0 = No bleeding within 30 s of probing;

1 = Bleeding within a few seconds of probing;

2 = Immediate bleeding on probing;

3 = Bleeding along the gingival sulcus at the slightest touch.

The Souto scale was chosen for its ability to assess post-surgical bleeding severity and the efficacy of hemostatic strategies, particularly in anticoagulated patients. The Mühlemann scale evaluates gingival bleeding and inflammation, providing a standardized measure of adjacent tissue health. Together, these scales ensure a comprehensive, validated assessment of bleeding and healing processes.

### 2.13. Pain

Pain was assessed according to the visual analog scale (VAS), with 0 indicating no pain and 10 indicating the most severe imaginable pain, together with the graphic rating scale [34]. After 1, 2, 3, and 7 days of extractions, at around 9 a.m., the participants self-reported the VAS by completing the evaluation form throughout the experimental periods.

### 2.14. Soft Tissue Healing

**Figure 4:**
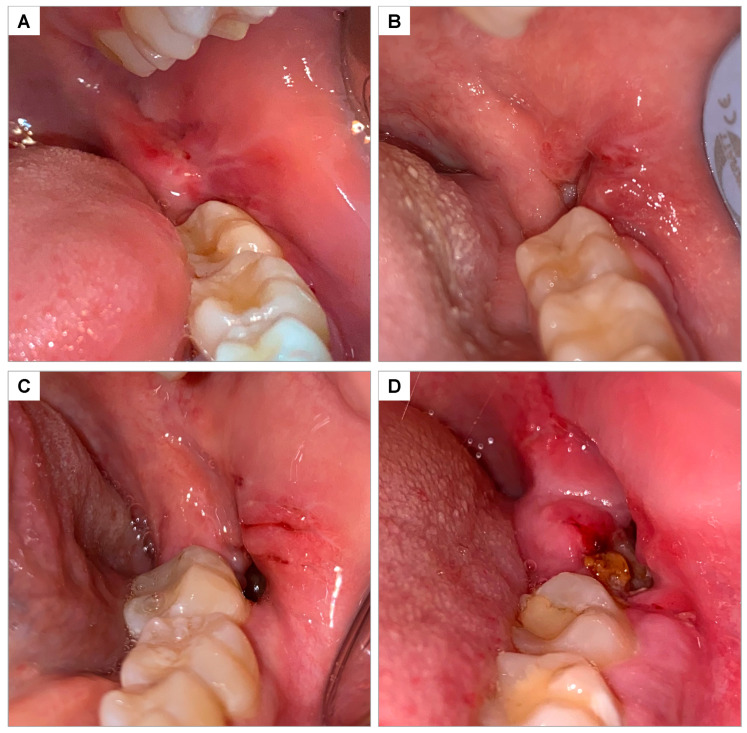
Clinical photograph illustrating the soft tissue healing scale according to Brancaccio et al., 2020 [35]: (**A**) score 0—complete closure without fibrin; (**B**) score 1—complete closure with fibrin; (**C**) score 2—incomplete closure of the socket (dehiscence); (**D**) score 3—incomplete closure with signs of necrosis.

The healing of soft tissues in the operated region was evaluated 7 and 14 days after extractions and classified on a scale of 0 to 3, according to Brancaccio et al., 2020 [35] (Figure 4):

0 = Complete closure without fibrin;

1 = Complete closure with fibrin;

2 = Incomplete closure of the alveolus (dehiscence);

3 = Incomplete closure with signs of necrosis.

### 2.15. Statistical Analysis

Calculations and graphs were created using Prism 10.0 (GraphPad Software, version 10.4.1). Fisher’s exact test was applied to analyze bleeding using the Souto and Muhlermann scoring methods, as well as repair processes. Pain analysis was conducted using the visual analog scale (VAS), expressed as median and 95% confidence intervals. The Kruskal–Wallis test was applied for comparisons, followed by Dunn’s post hoc test, with a significance level set at α = 0.05.

## 3. Results

**Figure 5:**
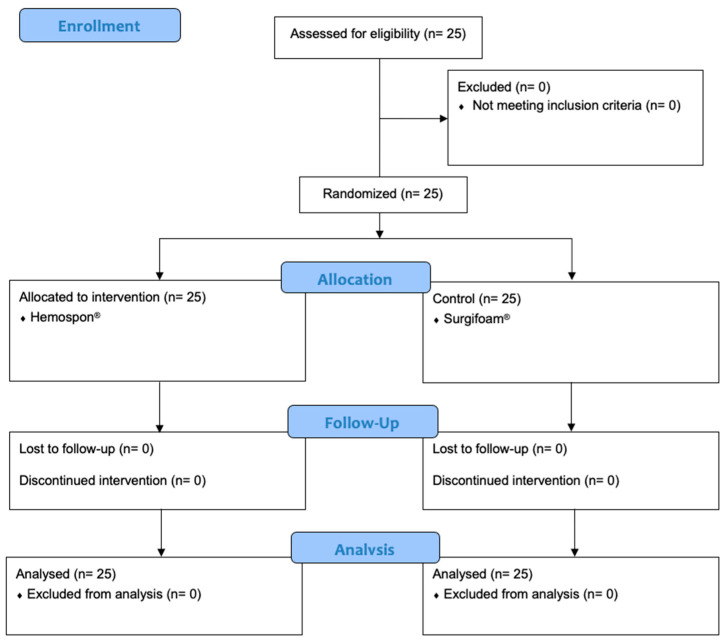
CONSORT flowchart.

**Figure 6:**
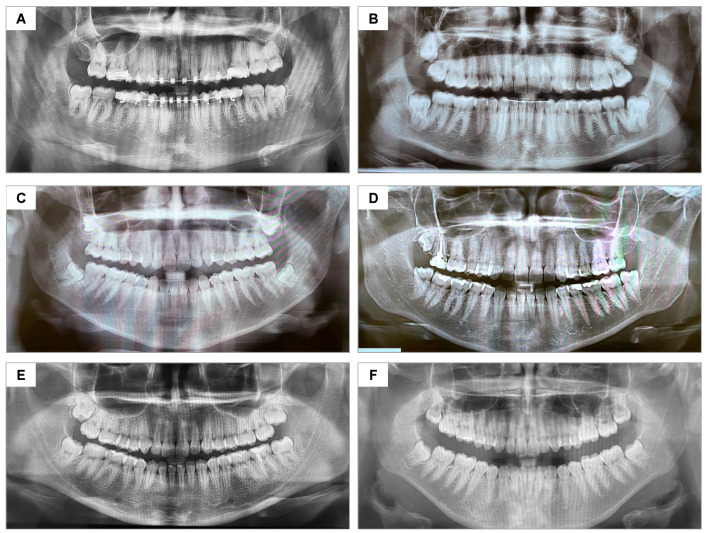
Panoramic radiographs representing the similarity in positioning and surgical difficulty of the lower third molars: (**A**) participant #18—A.G.S.J; (**B**) #7—M.P.M.B.; (**C**) #6—M.C.G.; (**D**) #4—J.A.A.L.; (**E**) #3—K.B.S.; (**F**) #11—B.V.R.

No dropout occurred, as shown in the CONSORT flowchart (Figure 5). No postoperative complications (e.g., infection, dry socket, or hemorrhage) were observed during evaluation periods. The control and test groups were considered homogeneous regarding the degree of difficulty of the extractions (Figure 6).

**Figure 7:**
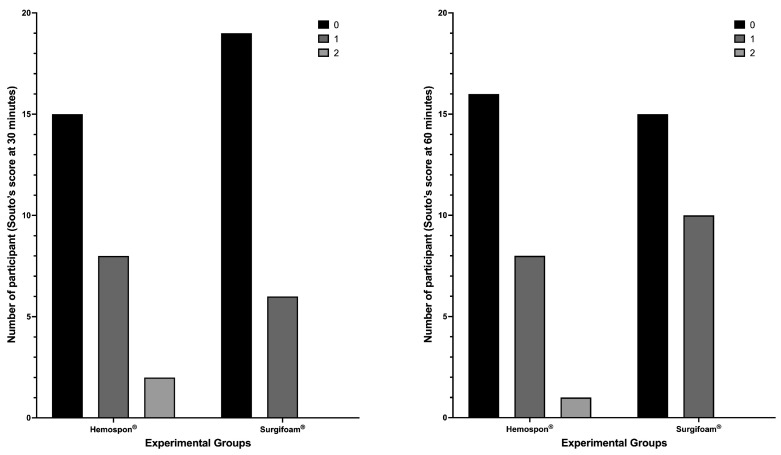
Bleeding assessment using the Souto scoring method at 30 and 60 min. The tested materials were Hemospon^®^ and Surgifoam^®^. The y-axis represents the number of participants who presented a given score (0, 1, or 2) as indicated in the legend. Fisher’s exact test was applied to evaluate significant differences between experimental groups, with α = 0.05.

The median bleeding scores and their respective 95% confidence intervals further support the observed trend in the distribution of bleeding scores. At 30 min, the median score for Surgifoam^®^ was 0 (95% CI: 0–1), while for Hemospon^®^, the median was also 0 (95% CI: 0–0), reinforcing the lack of significant differences between groups (*p* = 0.3157, Fisher’s exact test). Similarly, at 60 min, the median score remained 0 (95% CI: 0–1) for Surgifoam^®^ and 0 (95% CI: 0–1) for Hemospon^®^, suggesting comparable hemostatic effects over time (*p* = 0.7688, Fisher’s exact test). At 7 and 14 days, all participants exhibited a median score of 0 (95% CI: 0–0) in both groups, confirming complete hemostasis with no further bleeding events. Fisher’s exact test confirmed no significant differences between groups at these time points (*p* > 0.9999 for both; Figure 7).

**Figure 8:**
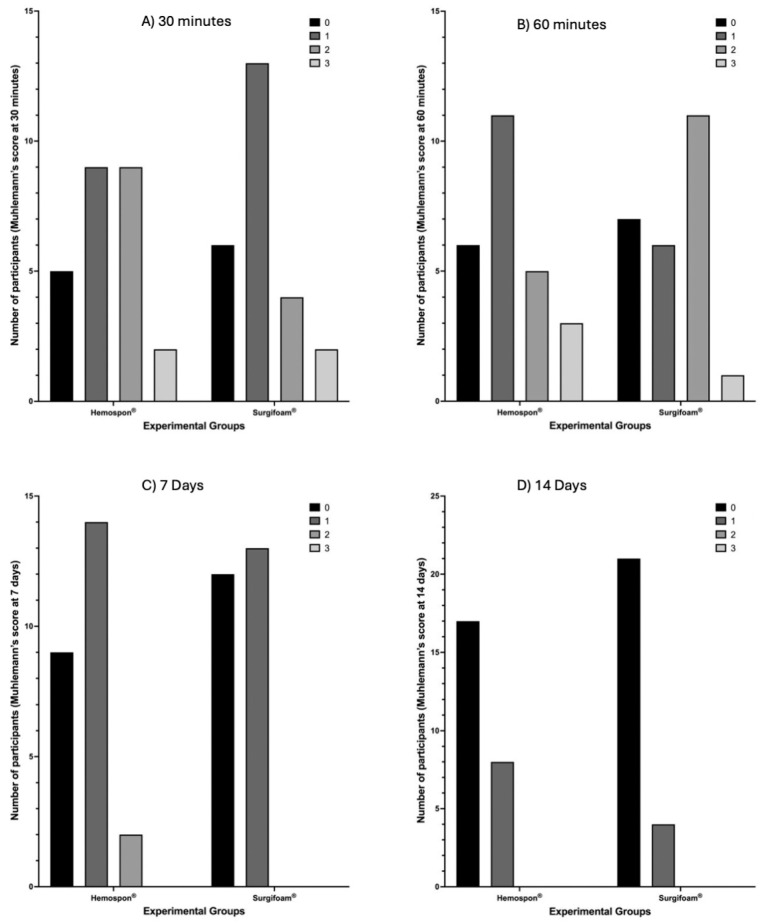
Bleeding assessment using the Muehlemann score at 30 min (**A**), 60 min (**B**), 7 days (**C**), and 14 days (**D**). The y-axis represents the number of participants with each score (0, 1, 2, or 3). Surgifoam^®^ showed a higher number of participants with no bleeding (score 0) at earlier time points, while Hemospon^®^ had more participants with moderate to severe bleeding (scores 2 + 3). Fisher’s exact test revealed no statistically significant differences between the groups at any time point (*p* = 0.4479; *p* = 0.2077, *p* = 0.4055, and *p* = 0.3209, respectively), with α = 0.05.

**Figure 9:**
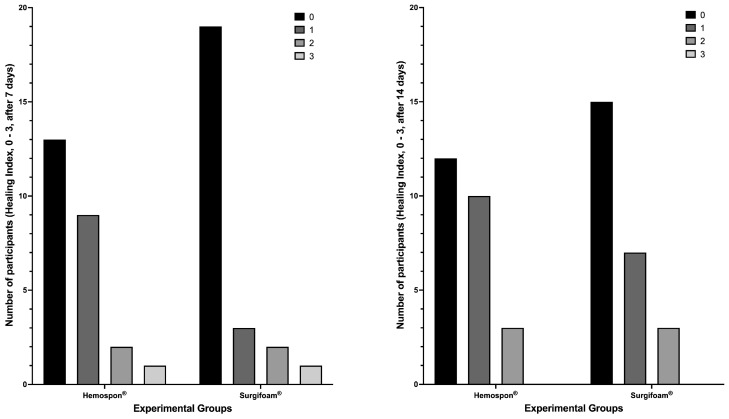
Soft tissue healing assessment at 7 and 14 days using the Brancaccio scale (scores 0–3). The y-axis represents the number of participants with each score (0, 1, 2, or 3). No significant differences were observed between the Hemospon^®^ and Surgifoam^®^ groups at 7 days (*p* = 0.200) and 14 days (*p* = 0.730) (Fisher’s exact test).

Using the Mühlemann score (Figure 8), Surgifoam^®^ had more participants with no bleeding (score 0) at both 30 and 60 min, while Hemospon^®^ showed higher frequencies of moderate to severe bleeding (scores 2 + 3). However, no statistically significant differences were observed (*p* = 0.4479 and *p* = 0.2077, respectively). The median Mühlemann score at 30 min was 1 (95% CI: 1–2) for Surgifoam^®^ and 1 (95% CI: 1–1) for Hemospon^®^, while at 60 min, the median remained 1 (95% CI: 1–2) for Surgifoam^®^ and 1 (95% CI: 1–1) for Hemospon^®^, indicating no major differences in hemostatic behavior between the two agents. By 7 and 14 days (Figure 9), nearly all participants in both groups scored 0, indicating resolution of bleeding, with no significant differences between ** Surgifoam^®^ and Hemospon^®^ (*p* = 0.4055 and *p* = 0.3209, respectively). The median score at 7 days for Surgifoam^®^ was 1 (95% CI: 0–2), while for Hemospon^®^, it was 1 (95% CI: 0–1), confirming minimal residual bleeding in both groups.

At 7 days, the healing index scores (0–3) showed that most participants in the Surgifoam^®^ group had higher scores (2 and 3), while the Hemospon^®^ group had a greater number of participants with a score of 0. Fisher’s exact test revealed no significant difference between the groups (*p* = 0.200). The median healing index at 7 days was 0 (95% CI: 0–1) for both Surgifoam^®^ and Hemospon^®^, suggesting a similar pattern of healing progression between the two groups. At 14 days, the distribution was similar, with Surgifoam^®^ presenting a higher proportion of participants with scores 2 and 3, while Hemospon^®^ maintained more participants with a score of 0. Fisher’s exact test confirmed no significant difference between the groups (*p* = 0.730). The median healing index at 14 days was 1 (95% CI: 0–1) for Surgifoam^®^ and 0 (95% CI: 0–1) for Hemospon^®^ (Figure 9).

**Figure 10:**
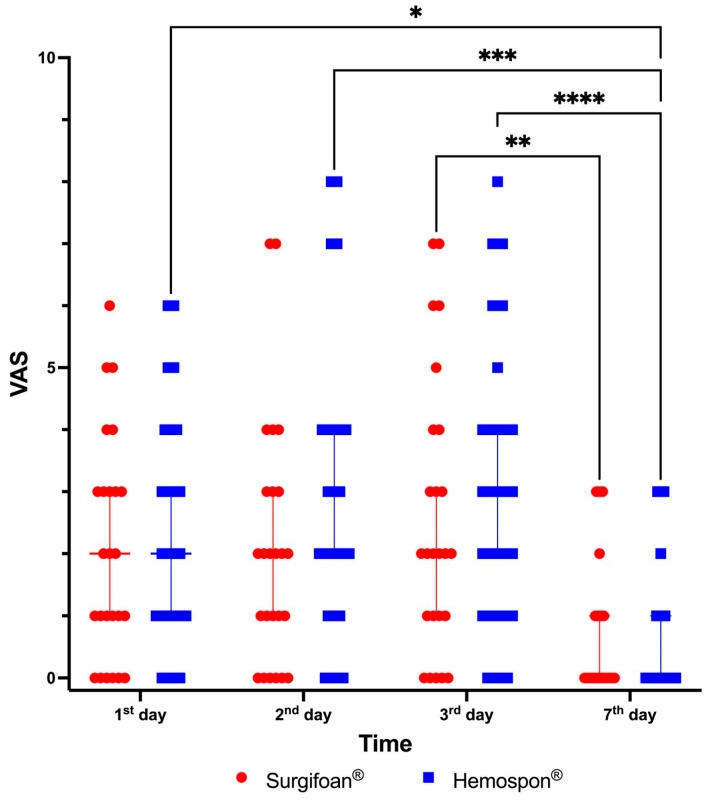
Postoperative pain evaluation using the Visual Analog Scale (VAS) at 1, 2, 3, and 7 days after tooth extractions. Data are expressed as individual VAS scores, along with each group’s median and 95% confidence intervals (Hemospon^®^ and Control—Surgifoam^®^). No significant differences were observed between groups within the same experimental periods (*p* > 0.05). Differences between the same treatment across different experimental periods are indicated * (*p* < 0.05), ** (*p* < 0.01), *** (*p* < 0.001), and **** (*p* < 0.0001, Kruskal–Wallis and Dunn’s test).

Figure 10 shows the results for evaluating postoperative pain according to the VAS. On the first day postoperatively, Visual Analog Scale (VAS) scores were highly variable across participants in both groups, with Surgifoam^®^ and Hemospon^®^ groups presenting a median VAS score of approximately 2 (range 1–6). By the seventh day, both groups showed a significant reduction in pain levels, with most participants reporting minimal or no pain (median VAS score 0 for both groups). The reduction in VAS scores between the first and seventh days was statistically significant (Figure 10). Despite these reductions, no significant differences in pain levels were observed between Surgifoam^®^ and Hemospon^®^ at any time point (*p* > 0.999).

## 4. Discussion

In oral surgery, complications such as pain, edema, trismus, and delayed healing from third molar extractions can significantly affect a patient’s quality of life [4,5,13]. A common technique to mitigate post-surgical complications is the use of collagen sponges, which promote wound healing, stabilize clots, and facilitate hemostasis by attracting fibroblasts to form a collagen scaffold [13,36].

This study evaluated the effectiveness of Hemospon and Surgifoam hemostatic sponges after lower third molar extractions, focusing on bleeding levels, soft tissue healing, and postoperative pain. Perioperative hemorrhage remains a major concern in dental surgeries, typically managed through direct pressure, electrocautery, or vessel ligation [35]. In certain cases, hemostatic agents are necessary adjuncts.

Complications associated with gelatin sponges, such as abscesses or granulomas, are rare [37]. According to Mani and Ebenezer, collagen sponges are non-toxic and absorbent, generally retaining their weight in fluid [38]. They are effective for wound protection and bleeding control, typically applied for 2 to 5 min before removal or left in situ. These sponges are reabsorbed within 14 to 56 days [36,37].

In this study, the sponges were effective in stabilizing blood clots without being visually detectable over time. While Mani and Ebenezer discussed the benefits of hemostatic agents [38], this study did not require clot control material. Surgical sites filled with Surgifoam exhibited low bleeding scores on the Souto (0–2) and Muehlemann (0–3) scales, showing no significant difference in bleeding between Surgifoam and Hemospon.

To assess pain levels, we used the VAS, rated by the participants to indicate pain intensity from zero (no pain) to 10 (maximum pain) [34,39,40]. Participants recorded pain levels at 1-, 2-, 3-, and 7-days post-extraction, revealing no significant differences between groups. This aligns with Kim et al., who noted improved tissue healing and reduced pain with hemostatic sponge use. Their study reported a VAS score of 1.42 ± 1.26 for the collagen sponge group versus 3.85 ± 2.43 for the control group 1-week post-surgery (*p* < 0.05).

While Kim et al. included antibiotics, their necessity in third molar extractions is debated. Gbotolorun et al. found no significant benefits in healthy patients [41], while Arteagoitia et al. noted amoxicillin did not reduce infection risk after extractions [42]. This study opted against antibiotics, prescribing analgesics and anti-inflammatories. It yielded similar results to Kim et al. regarding the absence of infections and alveolitis [13].

Kilinc observed no significant difference in pain between groups (*p* > 0.05). However, lower swelling was found in the second intention healing group (*p* = 0.046 and 0.00), and wound dehiscence occurred in 20% of the first intention group and 6.7% of the membrane-filled group. No infections or postoperative bleeding were recorded in 25 participants across 50 surgical sites [43].

Numerical analysis of the results highlights the similarity between the two groups. Pain scores on the first postoperative day showed a median of 2 (range: 1–6) for both Surgifoam and Hemospon groups, decreasing to a median of 0 by day 7. Bleeding scores at 30 min predominantly showed no bleeding (score 0) in 80% of participants using Surgifoam, compared to 60% in the Hemospon group, although the difference was not statistically significant (*p* > 0.05). Similarly, healing scores at 14 days revealed complete healing (score 0) in 90% of participants in both groups. These specific values reinforce the equivalence of the two agents.

Despite the advantages of hemostatic materials, gelatin sponges have limitations, such as low traction and adhesion capacity, weakening their hemostatic effectiveness [16]. Not all agents are suitable; potential risks include allergic reactions and infection [37]. Collagen sponges are contraindicated in contaminated sites due to the risk of bacterial growth [16,37].

Osteotomy may be necessary for tooth extraction in some cases, potentially exacerbating postoperative symptoms due to required bone remodeling [30,44]. All the study participants met strict inclusion criteria, ensuring consistent tooth positioning in relation to the second molar.

The lack of significant differences between Surgifoam and Hemospon aligns with prior studies, such as Nazari et al. [24], which demonstrated comparable hemostatic efficacy and biocompatibility between Gelfoam and Hemospon in animal models. Similarly, Kim et al. [13] found that collagen-based hemostatic sponges were effective in reducing pain and promoting healing, supporting our findings of equivalent outcomes for both agents. However, while Kilinc and Ataol [43] reported enhanced healing outcomes with the use of membranes in surgical wounds, our study did not observe such differences for these hemostatic sponges. This discrepancy may be attributed to differences in study design, follow-up duration, or the specific surgical contexts evaluated.

In conclusion, collagen sponge insertion after impacted lower third molar extraction may reduce early-stage postoperative complications. This effect is probably due to the role of the collagen sponge as an extracellular matrix, enhancing healing by promoting tissue maturation, revascularization, and fibroblast activity [13,45].

From a practical perspective, these findings suggest that both Surgifoam and Hemospon are effective options for bleeding control, promoting healing, and minimizing postoperative pain in oral surgery. Their comparable performance supports their use in clinical practice, allowing surgeons to select either agent based on factors such as availability and ease of use. Their application may be particularly beneficial in cases where achieving rapid hemostasis is critical, such as anticoagulated patients or those undergoing complex surgical extractions.

This study has some limitations that must be acknowledged. The small sample size may limit the generalizability of the findings, and the short follow-up period precluded the evaluation of long-term outcomes, such as bone regeneration or tissue stability. Additionally, although randomization and blinding were employed to minimize bias, potential residual biases cannot be entirely excluded. Further research with larger cohorts and extended follow-up durations is necessary to validate these findings.

Future research should explore the efficacy of Surgifoam and Hemospon in larger-scale randomized clinical trials, as well as in diverse surgical contexts such as periodontal surgeries or implant placement. Comparative studies involving other hemostatic agents, particularly those utilizing advanced biomaterials, may provide further insights into optimizing hemostatic strategies. Additionally, investigating long-term effects, including bone regeneration and patient satisfaction, could enhance the understanding of these agents’ broader clinical impact.

## 5. Conclusions

This study demonstrates that Surgifoam and Hemospon are both effective hemostatic agents for managing postoperative bleeding, promoting healing, and reducing pain in mandibular third molar extractions. Their comparable performance provides flexibility in clinical decision-making, allowing clinicians to choose based on factors such as availability or specific patient needs.

In settings where cost or accessibility differ, either agent can be confidently utilized without compromising clinical outcomes. These findings contribute to the evidence supporting hemostatic agent use in oral surgery and encourage further research in larger cohorts and diverse surgical contexts to expand their applications.

## Data Availability

The original contributions presented in the study are included in the article; further inquiries can be directed to the corresponding author.
